# Hexokinase 2, Glycogen Synthase and Phosphorylase Play a Key Role in Muscle Glycogen Supercompensation

**DOI:** 10.1371/journal.pone.0042453

**Published:** 2012-07-31

**Authors:** José M. Irimia, Jordi Rovira, Jakob N. Nielsen, Mario Guerrero, Jørgen F. P. Wojtaszewski, Roser Cussó

**Affiliations:** 1 Department of Physiological Sciences I, Institut d'Investigacions Biomediques August Pi i Sunyer (IDIBAPS), Universitat de Barcelona, Barcelona, Spain; 2 Molecular Physiology Group, Copenhagen Muscle Research Centre, Department of Exercise and Sport Sciences, University of Copenhagen, Copenhagen, Denmark; Université Joseph Fourier, France

## Abstract

**Background:**

Glycogen-depleting exercise can lead to supercompensation of muscle glycogen stores, but the biochemical mechanisms of this phenomenon are still not completely understood.

**Methods:**

Using chronic low-frequency stimulation (CLFS) as an exercise model, the *tibialis anterior* muscle of rabbits was stimulated for either 1 or 24 hours, inducing a reduction in glycogen of 90% and 50% respectively. Glycogen recovery was subsequently monitored during 24 hours of rest.

**Results:**

In muscles stimulated for 1 hour, glycogen recovered basal levels during the rest period. However, in those stimulated for 24 hours, glycogen was supercompensated and its levels remained 50% higher than basal levels after 6 hours of rest, although the newly synthesized glycogen had fewer branches. This increase in glycogen correlated with an increase in hexokinase-2 expression and activity, a reduction in the glycogen phosphorylase activity ratio and an increase in the glycogen synthase activity ratio, due to dephosphorylation of site 3a, even in the presence of elevated glycogen stores. During supercompensation there was also an increase in 5′-AMP-activated protein kinase phosphorylation, correlating with a stable reduction in ATP and total purine nucleotide levels.

**Conclusions:**

Glycogen supercompensation requires a coordinated chain of events at two levels in the context of decreased cell energy balance: First, an increase in the glucose phosphorylation capacity of the muscle and secondly, control of the enzymes directly involved in the synthesis and degradation of the glycogen molecule. However, supercompensated glycogen has fewer branches.

## Introduction

Glycogen, the branched polymer of glucose, is the main energy reserve in muscle and is also important for glucose homeostasis. Muscle glycogen levels are regulated by glycogenin, muscle glycogen synthase (mGS) and glycogen phosphorylase (mGPh). mGS is the key enzyme for glycogen synthesis. Regulation of its activity includes allosteric regulation by the activator glucose-6P and phosphorylation at nine different sites, which in general inactivates the enzyme [1]. Phosphorylation of sites 2 (Ser7 in the rabbit enzyme) and 2a (Ser10) in the N-terminus and sites 3a (Ser640) and 3b (Ser644) in the C-terminus has the greatest influence on its activity [2]. The phosphorylation state of the remaining sites (1a, 1b, 3c, 4 and 5) has little or no effect on enzyme activity *in vitro*.

Muscle glucose uptake and phosphorylation also control glycogen synthesis through substrate availability [3]–[5]. Glucose is phosphorylated by hexokinase. The vast majority of hexokinase activity in muscle is delivered by the hexokinase 2 isoform [6]. The 5′-AMP-activated protein kinase (AMPK) acts as a sensor of cellular energy charge [7]. It is activated by phosphorylation under conditions of energetic stress, such as muscle contraction. The enhanced activity of AMPK during contraction leads to increased muscle contraction-dependent glucose transport via an increase in GLUT4 recruitment to the cell membrane [3]. Clearly AMPK play a central role in muscle adaptation to contraction [8] and regulating glycogen synthesis through acute effects such as glucose availability and chronic effects involving the control of gene expression [9].

An increase in muscle glycogen content above basal levels is a well-established phenomenon known as supercompensation. It is widely used as a strategy by athletes to enhance performance in endurance events, as it helps to postpone fatigue [10]. It has been shown [11] that a high carbohydrate diet following glycogen-depleting exercise leads to supercompensation of muscle glycogen stores, which remain stable above basal levels for up to several days. Supercompensation can be achieved in other situations such as refeeding after starvation [12], chronic low-frequency stimulation [13], or in cultured muscle cells subjected to hypoxia [14]. Despite significant efforts to study this phenomenon, its mechanisms are still not fully understood. Various studies have suggested multiple mechanisms for glycogen supercompensation, such as altered glucose transport [15], increased availability of glycogenin [16], or reduced glycogen degradation by mGPh [13].

A better understanding of glycogen resynthesis and the mechanism(s) that lead(s) to supercompensation after muscle contraction is important not only to improve exercise performance in athletes, but also to understand the regulation of muscle glycogen metabolism and the role it plays in diseases such as type 2 diabetes. Using chronic low-frequency stimulation (CLFS) of rabbit muscle as an exercise model [17], a number of biochemical parameters related to glycogen metabolism and supercompensation were analyzed.

We report that the supercompensation mechanism is dependent on the overexpression of hexokinase 2 and coordinated changes in mGS, mGPh and AMPK activity. Together, these adaptations lead to a transient increase in muscle glycogen stores, composed of less frequently branched, enlarged molecules.

## Materials and Methods

### Chemicals and reagents

UDP-[U-^14^C]-glucose for use in glycogen synthase (GS) and glycogenin activity assays was synthesized as described previously [18]. Secondary antibodies coupled with horseradish peroxidase were purchased from Dakocytomation (Glostrup, Denmark). All other reagents were of analytical grade and were purchased from Sigma-Aldrich (Madrid, Spain) or Amersham Pharmacia Biotech (Munich, Germany), unless otherwise specified.

### Experimental model

**Figure 1:**
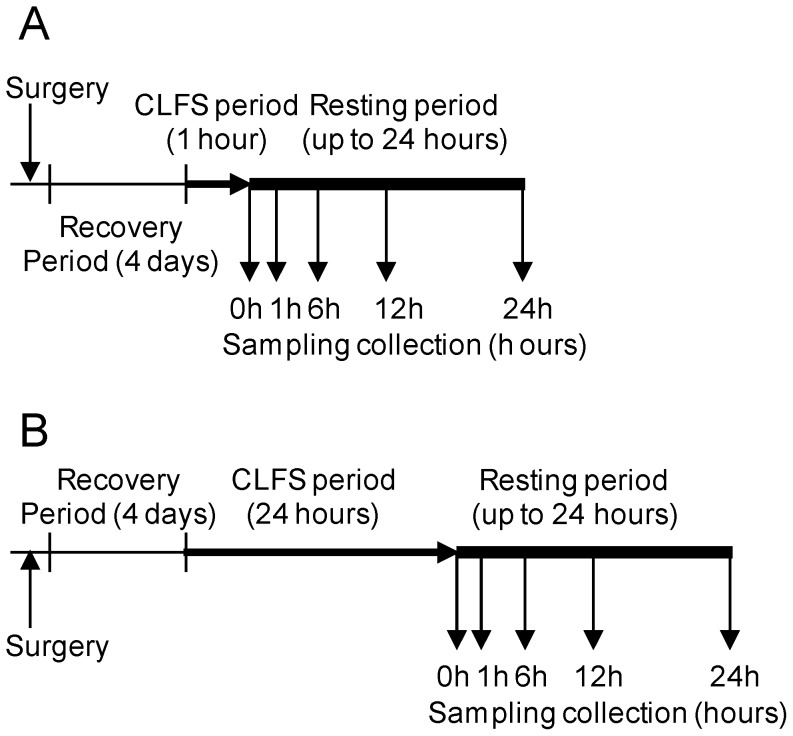
Experimental model. The horizontal arrows show the Chronic Low Frequency Stimulation period 1 hour (**A**) or 24 hours (**B**) after recovering from the surgery (4 days). Then both stimulated and non-stimulated contralateral *tibialis anterior* muscles were sampled at 0 hours (right after CLFS), 1, 6, 12 and 24 hours of rest. Collection of the samples was done always at the same hour of the day for both protocols.

All experiments were approved by the University of Barcelona Ethics Committee and complied with the APS's Guiding Principles in the Care and Use of the Animals. All surgical procedures were done under ketamine anesthesia and during the post-surgery recovery buprenorphine was administered to the animals. All efforts were done to minimize suffering. Adult female white New Zealand rabbits weighing between 3.0 and 3.5 kg were housed in temperature- and humidity-controlled conditions, with a 12 hour light/12 hour dark cycle. They were allowed food and water *ad libitum*. Comparable food consumption in all animals in different treatment groups was confirmed by weighing the food they had eaten. The composition of the diet (Teklad global 2030, Harlan, Indianapolis, IN) was 56.7% carbohydrate, 16.9% protein and 3% fat. Following the method described by Pette et al. [19], stimulating electrodes were implanted on both sides of the peroneal nerve, which innervates the fast-twitch *tibialis anterior* muscle of the left leg. Two electrostimulation protocols (10 Hz, 18 mA of amplitude and impulse width of 0.15 ms) were applied (Figure 1): 1 hour stimulation (1A), or 24 hours stimulation (1B). After stimulation, animals were allowed to recover for 0 (no rest), 1, 6, 12 or 24 hours. Following this recovery period, *tibialis anterior* muscles were taken and immediately frozen in liquid nitrogen and stored at −80°C until further analysis. To minimize circadian variability, all samples were collected between 12 pm and 2 pm by planning the start of the stimulation period accordingly. Five animals were sampled per group, collecting both the stimulated muscle (left leg) and the contralateral non-stimulated control (right leg), with n = 50 control samples in total. The insulin level in the serum was measured by radioimmunoassay (*Insulin-CT* kit, CIS Bio International). The glucose level in the serum was measured using a glucometer (*Lifescan OneTouch Ultra*).

### Metabolite measurement

Glycogen was extracted by digesting the tissue with 30% KOH, followed by three 66% ethanol precipitations as described previously [20]. The precipitate was digested and the glucose assayed as described by Prats et al. [17]. Krisman's method was used to assess glycogen branching [21]. In brief, extracted glycogen was mixed with a solution of saturated CaCl_2_ containing 6 mM KI and 0.4 mM I_2_. The absorbance spectrum was recorded from 350 to 750 nm. Extraction of metabolites and nucleotides was performed as described elsewhere [22]. ATP, creatine, creatine-P, glucose and glucose-6P were assessed as described previously, following the method of Lowry and Passonneau [23]. Purine nucleotide determination (ATP, ADP, AMP, Adenosine, IMP, and NAD) was performed by reverse-phase high performance liquid chromatography (HPLC), following the method of Tullson et al. [24]. The AMP/ATP ratio and total purines were calculated using the ATP data obtained via HPLC.

### Enzyme activities

All enzyme activity assays were conducted at 30°C. Fractional GS activity was measured in total extracts following the method described by Guinovart et al. [25]. GPh activity was measured following the method of Gilboe et al. [26]. The latter method was also used to elongate glycogen. Briefly, the reaction contained citrate 100 mM (pH 7.4), AMP 5 mM, purified GPh b in excess (0.2 mg/ml), glucose-1P 50 mM and purified glycogen as the limiting substrate (up to 10 micrograms/ml). Glycogen was purified following the method of Tagliabracci et al. [27]. Total hexokinase (HK) activity was measured as described by Davidson et al. [28] using 0.1% Triton X-100 in the extraction buffer. Glycogenin transglucosylase activity was measured following the method of Cao et al. [29], with some modifications. Briefly, the homogenate was treated with α-amylase 40 U/ml for 1 hour at room temperature or left untreated and immediately assayed. Five microlitres of homogenate was added to 5 µl of freshly prepared reaction mix and assayed as described above. The activity in each sample was taken as the difference between activity in the amylase-treated sample and that in the untreated sample. Glycogen branching enzyme (GBE) activity was assayed indirectly by measuring the stimulation of incorporation of glucose-1-P into glycogen by glycogen phosphorylase b as described by Bruno et al. [30]. Sufficient purified glycogen was added to the reaction to avoid artifacts due to differences in endogenous glycogen. Protein concentration was measured using the Bradford method [31].

### Immunoblot analysis

The primary antibodies used were: muscle GS (mouse monoclonal from Chemicon, dilution 1∶2000), glycogenin (guinea pig polyclonal, dilution 1∶2000), AMPK-P (T172) (rabbit polyclonal from Cell Signalling, dilution 1∶1000), and AMPKα1 or AMPKα2 (goat polyclonal, dilution 1∶2000). Antibodies against GS phosphorylated at sites 1a (Ser697), site 1b (Ser710), 2, 2+2a (Ser7 and Ser10), 3a (Ser640), 3b (Ser644), 4 and 4+5 (Ser652 and Ser656) were raised in goat, and were used at a dilution of 1 µg/ml (sites 3a, 3b, 4 and 5), 2 µg/ml (site 2+2a), 1∶700 (site 2), 1∶1000 (site 1b) or 1∶2000 (site 1a), as previously described [32]. Immunoblots were performed as described previously [17]. Phosphorylated AMPK content was normalized by AMPKα1 total content, while phosphorylation of the different sites of mGS was normalized by mGS total protein content. Glycogenin, total GS, AMPKα1 and α2 content were normalized by total protein measured in Coomassie brilliant blue stained gels (linear from 1 to 30 micrograms of protein). This method was used to avoid artifacts due to differences in other housekeeping proteins, the content of which may change according to the stimulation protocol.

### RNA isolation and quantitavive PCR

RNA was prepared with the TriPure Isolation Reagent® (Roche), following the procedure suggested by the manufacturer. The RNA was then subjected to reverse transcription using the Superscript II-RT kit (Invitrogen) according to the manufacturer's instructions. The cDNA was stored at −80°C before subsequent analysis. Primers and probes were designed for the *hk2* rabbit gene, according to the requisites of Taqman real-time PCR using the program Primer Express® (Applied Biosystems): TGCTGCCGACCTTTGTGA as the forward primer, AAGGTCCAGAGCCAGGAACTC as the reverse primer, and TCCACTCCAGATGGCACAGAACACG as the probe. The probe was labeled with FAM as the dye and TAMRA as the quencher. 18S rRNA was used as a housekeeping control gene (assay-on-demand® hs99999901_s1, Applied Biosystems). The Real-Time PCR protocol was performed according to the manufacturer's instructions (Applied Biosystems). The fold-change in the *hk2* mRNA content of stimulated samples with respect to the contralateral non-stimulated control samples was calculated following the method of Pfaffl [33].

### Statistical Analyses

For each parameter, kurtosis and skewness were calculated to test for a normal distribution. Normally distributed data obtained from the stimulated muscle were compared against data obtained from the contralateral non-stimulated control muscle using Multiple-Way Analysis of Covariance, followed by the LSD post-hoc test. For studies in which only a number of control muscles were assayed, groups were compared using a Multiple-Way Analysis of Variance, followed by the LSD post-hoc test. In non-normally distributed datasets, the values of all groups were compared using the non-parametric Mann-Whitney *U*-test and the Kruskal-Wallis test.

## Results

### Glycogen content and structure

**Figure 2:**
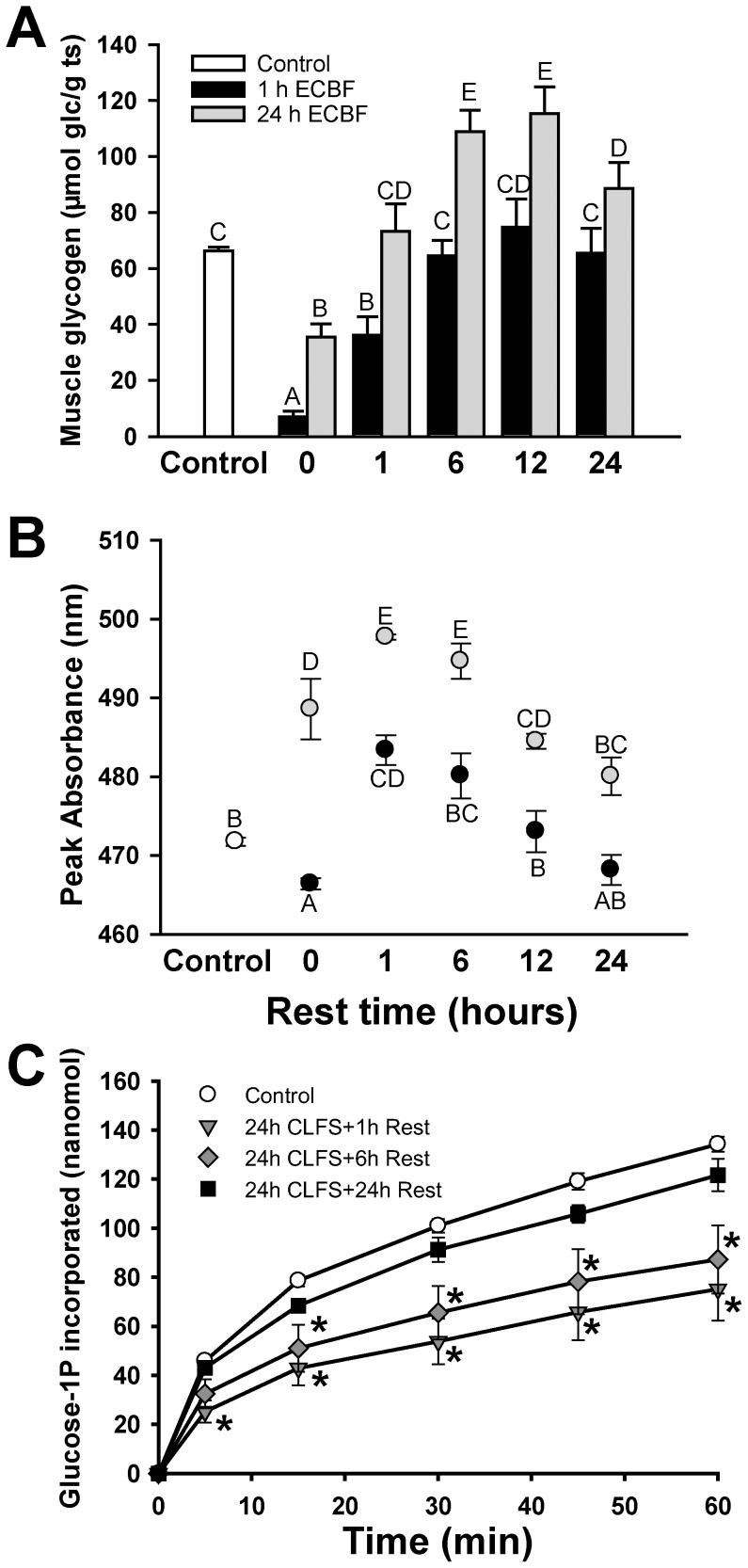
Glycogen content and the absorbance branching index. (**A**) Glycogen content of 1 hour (▪ bar) or 24 hours (▪ bar) stimulated muscles (µmols of glucose/g tissue). (**B**) Wavelength of the maximum absorbance peak (nm) as branching index (▪ dots are 1 hour CLFS samples and ▪ dots are 24 hours CLFS samples). Control group (□ bar or dot) was composed with the pool of all contralateral non-stimulated muscle samples results (n = 50). n = 5 for the stimulated and rested groups. (**C**) The same amount (2.4 micrograms) of control (white circles), poorly branched normal content (24 h CLFS+1 h rest, grey triangles), poorly branched supercompensated (24 h CLFS+6 h rest, grey diamond) and normal branched supercompensated (black squares, 24 h CLFS+24 h rest) purified glycogen samples were elongated using GPh-b. Glucose incorporation from G1P was monitored at 5, 15, 30, 4 and 60 minutes. n = 3–5. Data are means ± SE. Bars or dots with the same letter are not significantly different from each other. *: p<0.05 compared with control.

After 1 hour of CLFS there was a significant reduction in glycogen content, reaching 10% of the basal value (Figure 2A). Glycogen levels recovered to levels comparable to those in the control muscle after 6 hours of rest. After 24 hours of CLFS, glycogen decreased to 50% of the control. During the subsequent rest period, levels reached basal values after 1 hour, and then further increased up to 6 hours, leading to supercompensation. Levels then remained stable for up to 24 hours of rest. We then analysed the degree of branching by intercalating iodine in the purified glycogen (Figure 2B). A shift in the absorption maximum to a higher wavelength correlates with a lower degree of branching. After 1 hour of CLFS, and coinciding with the lowest glycogen levels, branching increased, and then decreased during the glycogen resynthesis period, progressively returning to basal values. After 24 hours of CLFS, glycogen branching was significantly lower than normal. The lowest branching value was observed during resynthesis and supercompensation. Normal branching values were only reached after 24 hours of rest. To test the effect of branching on the efficiency of glycogen as a substrate, 2.4 micrograms of purified glycogen extracted from samples under different situations were elongated using glycogen phosphorylase and glucose-1-P in excess (Figure 2C). Incorporation of glucose into poorly branched glycogen at basal levels (24 h CLFS +1 h rest) or supercompensated levels (24 h CLFS+6 h rest) was reduced, but did not differ from the control in normally branched supercompensated glycogen (24 h CLFS+24 h rest).

### Blood glucose, insulin and muscle metabolite and nucleotide levels

There was no significant modification in blood insulin during CLFS or the rest period (Table 1). Blood glucose was slightly increased at the beginning of the rest period in both protocols, but subsequently returned to basal levels. Muscle glucose-6P (Table 1) levels decreased significantly after the period of stimulation (both 1 and 24 hours), returning to basal levels once glycogen had been resynthesized.

**Table 1:** Blood parameters, metabolites and nucleotide levels in muscle.

CLFS	Control	1 hour of CLFS					24 hours of CLFS				
Rest (hours)		0	1	6	12	24	0	1	6	12	24
Blood glucose	166±3	175±17	234±20 *	174±14	233±14 *	222±21	176±14	228±9 *	203±8	228±44	225±26
Insulin	32.0±4.8	23.4±3.7	42.2±12.7	37.7±16.2	41.5±12.1	36.8±8.6	62.3±20.9	35.4±13.3	55.7±20.0	51.4±16.0	31.6±5.0
Glc-6P	0.85±0.06	0.25±0.04 *	0.53±0.08 *	0.59±0.16 +	0.91±0.08 +	0.66±0.28 +	0.21±0.04 *	0.44±0.05 *	0.64±0.17 +	0.41±0.07 +	0.92±0.20 +
ATP(enz)	5.54±0.13	2.58±0.23 *	4.09±0.45 * +	6.00±0.47 +	5.11±0.26 +	5.30±0.73 +	4.32±0.23 * $	4.25±0.24 *	4.27±0.30 * $	4.80±0.25 *	4.57±0.16 *
ATP (hplc)	4.29±0.10	2.28±0.10 *	3.69±0.28 +	4.08±0.22 +	N.D.	N.D.	3.07±0.06 * $	3.45±0.11 *	3.31±0.10 * $	N.D.	N.D.
ADP	0.584±0.020	0.502±0.018 *	0.565±0.034	0.590±0.016	N.D.	N.D.	0.504±0.017 *	0.556±0.023	0.492±0.010 * $	N.D.	N.D.
AMP	6.24±0.67	7.58±1.88	8.16±1.53	8.17±1.55	N.D.	N.D.	6.04±0.72	6.86±1.27	2.75±0.36	N.D.	N.D.
Adenosine	2.8±0.6	18.5±6.4 *	36.5±2.5 *	13.3±3.2 *	N.D.	N.D.	36.7±2.6 *	17.9±5.8 *	29.7±2.6 *	N.D.	N.D.
IMP	0.025±0.011	0.247±0.043 *	0.023±0.005+	0.004±0.003 +	N.D.	N.D.	0.045±0.011 $	0.008±0.002	0.007±0.004	N.D.	N.D.
NAD	0.597±0.023	0.475±0.042 *	0.601±0.037+	0.618±0.025+	N.D.	N.D.	0.482±0.025 *	0.550±0.038	0.470±0.009 * $	N.D.	N.D.
Total Purines	4.91±0.12	3.06±0.12 *	4.32±0.31 +	4.69±0.23 +	N.D.	N.D.	3.66±0.07 *	4.04±0.13 *	3.84±0.11 * $	N.D.	N.D.
Ratio AMP/ATP	1.46±0.17	3.36±0.87 *	1.62±0.13 +	2.05±0.43 +	N.D.	N.D.	2.22±0.39	2.39±0.49	0.83±0.11 +	N.D.	N.D.
Total Creatine	34.5±1.3	33.3±1.9	36.4±1.9	37.5±2.1	35.9±1.2	33.9±1.4	29.5±1.0	34.8±2.6	35.8±1.5	36.6±1.1	37.1±1.4
Ratio P-Cr/Total Cr	0.74±0.01	0.60±0.03 *	0.81±0.02 * +	0.63±0.03 *	0.72±0.03 +	0.70±0.02 +	0.67±0.04	0.71±0.03 $	0.53±0.01 * $ +	0.63±0.04 * $	0.53±0.04 * $ +

Blood glucose is expressed as (mg/dl). Insulin is expressed as (mU/l). For these parameters control n = 17 (obtained from unstimulated animals) and stimulated groups n = 3–5. Glucose-6P, ATP, ADP, IMP, NAD, total purines and total Creatine content are expressed as (micromol/g tissue). Adenosine and AMP are expressed as (nanomol/g tissue). Ratio AMP/ATP is expressed as (nanomol AMP/(micromol ATP_hplc_). ATP (enz) data was obtained using the enzymatic assay, while ATP (hplc) values were obtained using HPLC. Control group was composed with the pool of 5–10 contralateral non-stimulated muscle samples. n = 5 for the stimulated and rested groups. Data are means ± SE.

* : p<0.05 compared with control.

$ : p<0.05 compared with 1 hour of CLFS samples with the same time of rest.

+ : p<0.05 compared with same CLFS time and 0 hours of rest.

In order to evaluate the energy status of the muscles, the ATP content (enzymatically and by HPLC), total purines (ATP+ADP+AMP+adenosine+IMP), the AMP/ATP ratio, total creatine and creatine-P/total creatine ratio were determined (Table 1). The results suggest that differences in ATP content were the determining factor in changes in total purines and the AMP/ATP ratio, so only ATP was measured in groups beyond 6 hours of rest. The ATP content was significantly reduced after 1 hour of CLFS but had recovered after 6 hours of rest. In contrast, although the levels of ATP were also significantly decreased after 24 hours of CLFS, 24 hours of rest were not sufficient to recover control values. The total creatine levels were unchanged in all situations, while the creatine-P/total creatine ratio generally followed a similar pattern to ATP levels.

### Glucose phosphorylation

**Figure 3:**
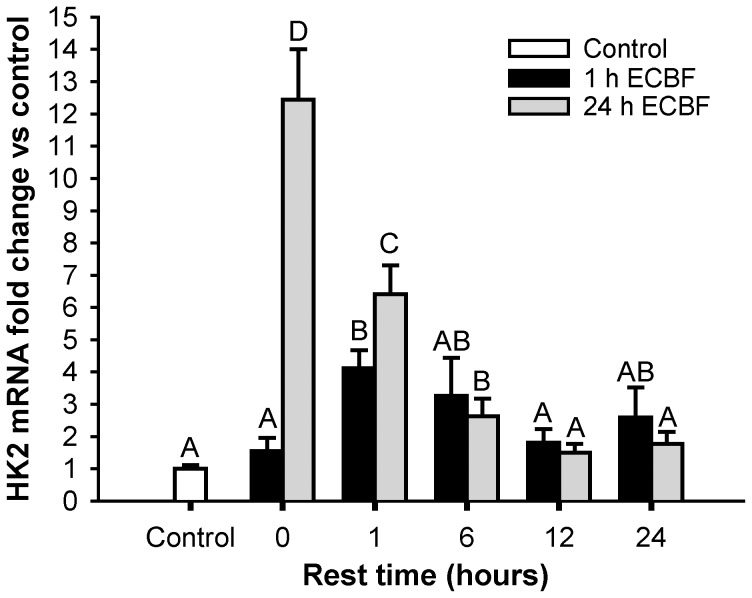
HK2 cDNA change. Fold change in *hk2* mRNA content of 1 hour (▪) or 24 hours (▪) stimulated samples compared to control situation measured with Real-Time PCR. Control group (□) was composed with the pool of all contralateral non-stimulated muscle samples results (n = 50). n = 5 for all the other groups. Data are means ± SE. Bars with the same letter are not significantly different from each other.

We then focused on glucose phosphorylation. After 12 hours of rest, HK activity (Table 2) increased significantly in the 1-hour CLFS group, preceded by an increase in hexokinase 2 mRNA (Figure 3). The 24-hour CLFS group showed a prominent and stable increase in HK activity throughout the resting period, accompanied by an even greater increase in the *hk2* transcript, which returned to control values after 12 hours of rest.

**Table 2:** Enzyme activities in skeletal muscle.

CLFS	Control	1 hour of CLFS					24 hours of CLFS				
Rest (hours)		0	1	6	12	24	0	1	6	12	24
HK	15.6±0.5	16.8±2.1	17.9±2.0	15.6±1.0	17.9±2.0 *	23.7±0.6 * +	24.3±1.2 * $	24.7±2.0 * $	25.1±3.0 * $	25.2±2.0 * $	28.0±0.8 * $ +
GNG	38.4±1.8	54.1±11.2	29.7±4.9	44.1±3.6	40.4±7.2	37.4±7.6	35.1±5.0	36.5±3.2	38.5±.8	47.1±2.7	36.7±5.7
Ratio GS-L/H	0.15±0.01	0.83±0.02 *	0.60±0.05 *	0.32±0.08 +	0.10±0.03 +	0.14±0.04 +	0.66±0.03 *	0.44±0.06 * +	0.12±0.05 $ +	0.14±0.03 $ +	0.06±0.01 +
GS-H	16.7±0.6	4.97±0.61 *	17.6±1.9 +	19.7±2.0 +	13.9±2.7 +	16.2±1.3 +	17.0±2.2 $	19.6±1.9	15.9±1.9	17.3±0.7	11.7±1.3
Ratio GPh-a/total	0.70±0.01	0.02±0.01 *	0.43±0.09 *	0.57±0.08 +	0.73±0.02 +	0.53±0.09 +	0.17±0.04 *	0.29±0.06 *	0.45±0.09 * $ +	0.42±0.04 * $ +	0.61±0.05 +
GPh total	2.48±0.08	1.87±0.07	2.70±0.25	2.29±0.16	2.19±0.05	2.18±0.23	2.40±0.23	2.13±0.09	2.41±0.24	2.47±0.25	2.37±0.22
GBE	0.491±0.031	0.512±0.036	0.593±0.016	0.397±0.016	0.575±0.104	0.481±0.009	0.409±0.015	0.552±0.056	0.518±0.067	0.462±0.057	0.715±0.024 * +

Enzyme activities are expressed as: hexokinase (HK) (Units/g protein), Glycogenin (GNG) (nanomol UDP-glucose/g protein · min), glycogen synthase total (GS-H) (nanomol UDP-glucose/mg protein · min), glycogen phosphorylase total activity (GPh total) and glycogen branching enzyme (GBE) (µmol Glucose-1-P/mg protein · min). Ratio GS-L/H is the ratio of the GS activity measured with low glc-6P (L) over GS measured with high glc-6P (H). Ratio GPh-a/total is the ratio of the GPh activity measured without (a) over with AMP (total). Data are means ± SE. Control group was composed with the pool of all contralateral non-stimulated muscle samples (n = 50), except for branching enzyme (n = 10). n = 5 for the stimulated and rested groups, except for branching enzyme (n = 3).

* : p<0.05 compared with paired control.

$ : p<0.05 compared with 1 hour of CLFS samples with the same time of rest.

+ : p<0.05 compared with same CLFS time and 0 hours of rest.

### Glycogen synthesis

**Figure 4:**
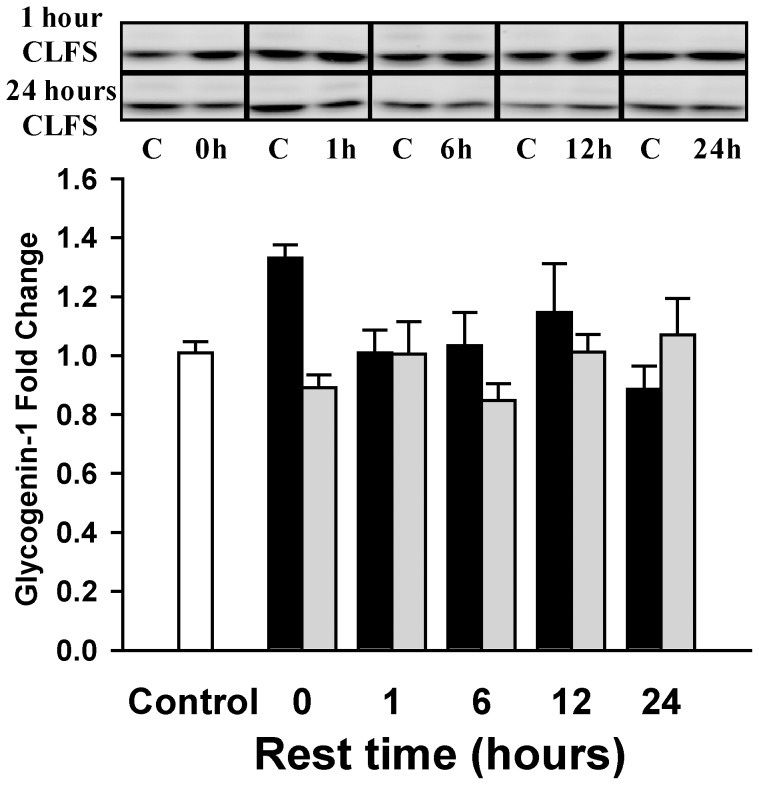
Change of glycogenin-1 protein content. Fold change in glycogenin normalized by total protein content of 1 hour (▪) or 24 hours (▪) stimulated samples compared to control situation (□) measured by Western-blot of Glycogenin-1. The inset shows a representative blot for each situation (from 0 to 24 hours of rest): in the upper panel samples with 1 hour of CLFS, and the lower panel the groups with 24 hours of CLFS. C is the contralateral non-stimulated control sample of each sample showed. In the bar chart the control group was composed with the pool of all contralateral1 non-stimulated muscle samples results (n = 50). n = 5 for the stimulated and rested groups. Data are means ± SE. There was no statistical difference under any condition.

**Figure 5:**
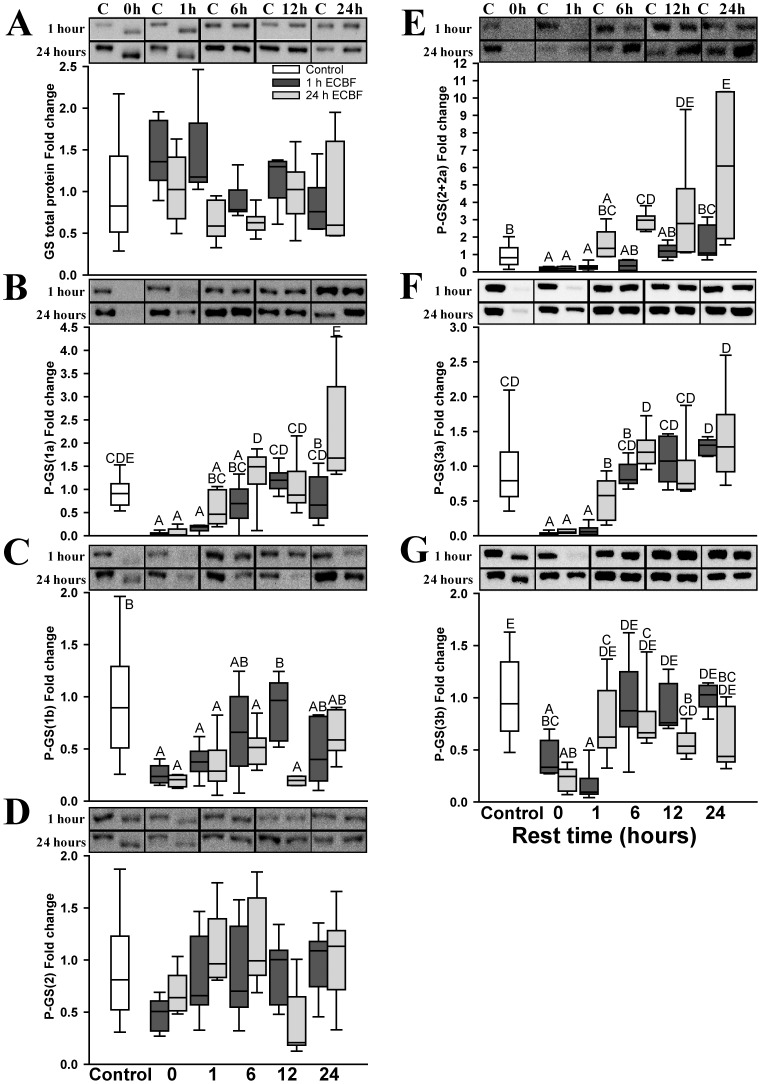
Total mGS protein and phosphorylation content of the different sites. Fold change of 1 hour (▪) or 24 hours (▪) stimulated samples compared to control situation (□) measured by Western-blot of: (**A**) Total mGS protein content normalized by total protein and phosphorylation of mGS in (**B**) site 1a, (**C**) site 1b, (**D**) site 2, (**E**) site 2+2a, (**F**) site 3a and (**G**) site 3b, normalized by mGS. The control was composed with the pool of all contralateral non-stimulated muscle samples results (n = 50). For the stimulated and rested groups n = 5. The upper panels of each chart contain representative blots to the total mGS or the phosphorylated sites: 1 hour CLFS the upper lane and 24 hours CLFS the lower lane. C: The correspondent non-stimulated control, and 0 h, 1 h, 6 h, 12 h and 24 h are the time of rest after the stimulation. Data are expressed as box and whisker plot with the median as a transversal line in the box. Groups with the same letter are not significantly different from each other.

We subsequently studied the enzymes controlling glycogen synthesis. There was no change in glycogenin transglycosylating activity (Table 2) or protein content (Figure 4) in any situation. Total mGS activity, measured by the presence of high glucose-6P (GS-H), remained unchanged (Table 2), with the exception of immediately after 1 hour of CLFS, when a decrease of about 70% was detected, although this was not correlated with the protein levels measured by Western blotting. mGS protein levels did not change significantly under any situation (Figure 5A). The L/H activity ratio (Table 2) increased after 1 hour of CLFS, returning to basal levels after 6 hours of rest, which coincided with the restoration of glycogen content. After 24 hours of CLFS, the activity ratio showed a less substantial increase, but remained high after 1 hour of rest, even though glycogen levels had already been restored. After 6 hours of rest, the activity ratio returned to basal values.

The phosphorylation content of the different sites of mGS was measured by Western blotting using phosphospecific antibodies to the sites 1a, 1b, 2, 2+2a, 3a and 3b (Figure 5). The phosphorylation of sites 1a, 1b, 2+2a, 3a and 3b was reduced during both CLFS events and after 1 hour of rest following 1 hour of CLFS, correlating with the reduction in glycogen content. The phosphorylation content of sites 1a (Figure 5B), 2+2a (Figure 5E) and 3b (Figure 5G) returned to normal values when glycogen stores achieved basal levels. Moreover, when glycogen was supercompensated site 2+2a was hyperphosphorylated. The phosphorylation content of site 1b (Figure 5C) and 3a (Figure 5F) followed the same pattern as sites 1a and 3b after 1 hour of CLFS. However, after 24 hours of CLFS the phosphorylation content of site 1b remained lower for up to 12 hours of rest and that of site 3a for up to 1 hour of rest, even though glycogen levels had already been restored. Both later returned to baseline levels. Site 2 (Figure 5D) phosphorylation remained unchanged in all situations.

GPh activity, measured in the presence of AMP (GPh total), was unchanged in all situations (Table 2). The GPh activity ratio (Table 2) decreased after 1 hour of CLFS, returning to basal levels as glycogen content was restored. The activity ratio decreased less drastically after 24 hours of CLFS compared to after 1 hour. It was still low after 12 hours of rest. The activity ratio only returned to basal values after 24 hours of rest.

Glycogen branching enzyme activity remained unchanged, except in the 24-hour CLFS/24-hours rest group, in which we recorded an increase in total activity of 45% over the control.

### AMPK

**Figure 6:**
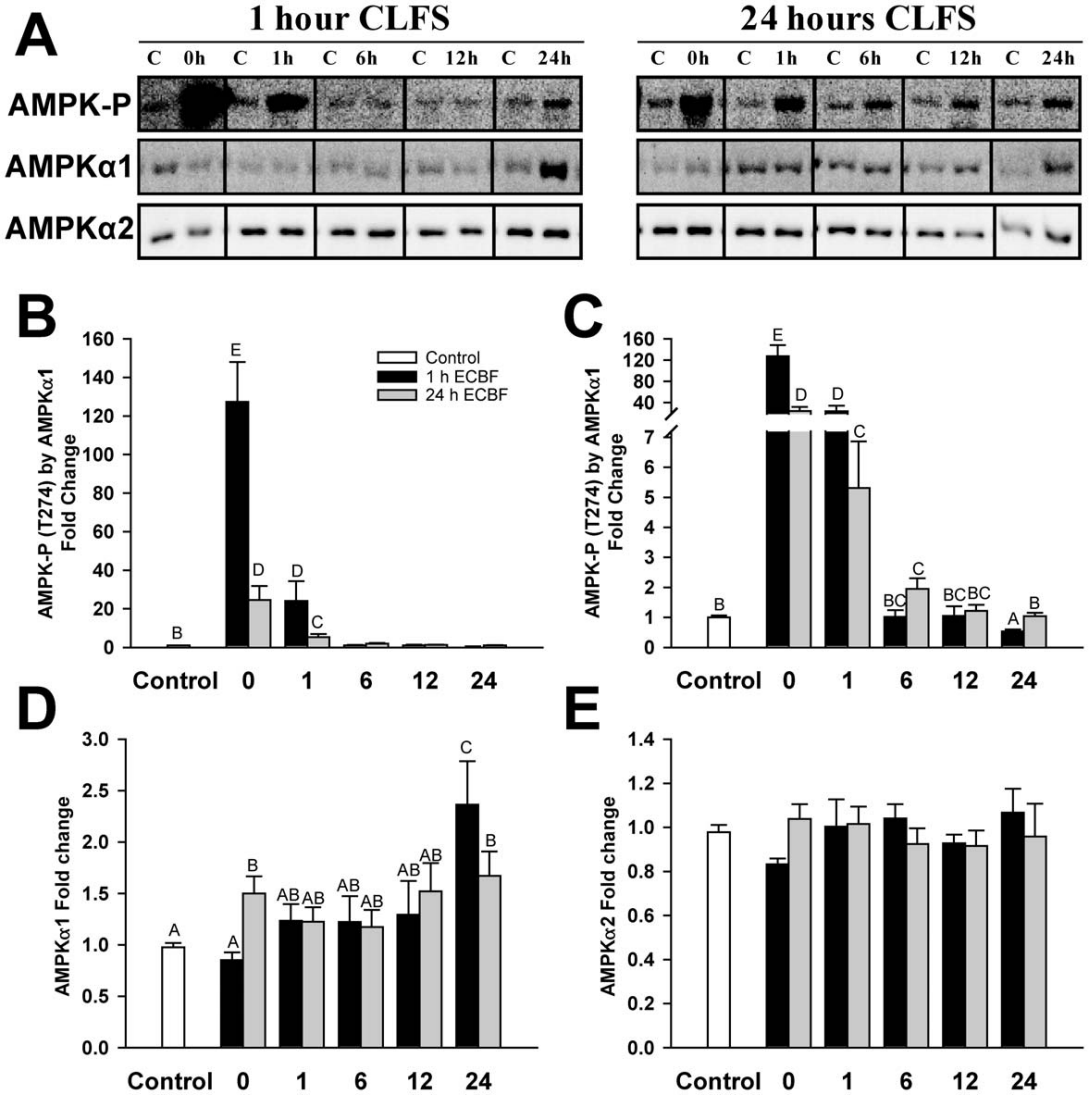
The activation state of AMPK. (**A**) Representative blots of AMPK-P (T172), AMPKα1 and AMPKα2 isoforms. 1 hour CLFS samples on the left and 24 hours CLFS on the right. Samples are C: The correspondent non-stimulated control, and 0 h, 1 h, 6 h, 12 h and 24 h are the time of rest after the stimulation. (**B**) Quantification of the AMPK-P (T172) Western-blot blot normalized by AMPKα1 in fold change (from 0 to 160 fold) compared to control and in a different scale (**C**) with a break from 7 to 35 to better display the data in the control samples and samples rested from 6 to 24 hours. Quantification of AMPKα1 (**D**) and AMPKα2 (**E**) in fold change compared to control from the Western-blot normalized by total protein content. 1 hour (▪) and 24 hours (▪) stimulated samples n = 5. Control group (□) was composed with the pool of all contralateral non-stimulated muscle samples results (n = 50). Data are means ± SE. Bars with the same letter are not significantly different from each other.

Lastly, we analyzed AMPK. The activation state of AMPK, as judged by phosphospecific antibodies to the activating T-loop phosphorylation site, showed that phosphorylation greatly increased after 1 hour of CLFS and, in parallel with the restoration of glycogen levels, returned to basal values after 6 hours of rest (Figure 6A, 6B, 6C). After 24 hours of CLFS, AMPK phosphorylation increased to a lesser extent than after 1 hour of CLFS, but the levels were only restored after 12 hours of rest, long after glycogen levels were restored. The levels of isoform AMPKα2, the catalytic subunit of AMPK that is phosphorylated, remained unchanged under all conditions (Figure 6E). However, the amount of isoform α1 (Figure 6D) significantly increased after 24 hours of rest in both protocols.

## Discussion

The aim of this study was to examine the response of glycogen metabolism to rest after the depletion of glycogen following 1 or 24 hours of CLFS. The results obtained after 1 hour of CLFS contrast with studies in which glycogen was supercompensated after glycogen depletion followed by a carbohydrate load [11], [34], [35], possibly because the animals were fed a diet that was moderately high in carbohydrates (56.7%) *ad libitum*. In contrast, after 24 hours of CLFS, rest led to clear supercompensation of glycogen above 50% of basal levels. Given that the eating behaviour, blood glucose and insulin levels were the same in both CLFS groups, we believe that the glycogen supercompensation mechanism in this model is dependent exclusively on local muscle effectors such as the stimulation and resynthesis conditions.

One of the major findings in this comparative study is that a sustained and time-coordinated decrease in cellular energy charge and increase in glucose phosphorylation activity and glycogen synthesis are required to allow glycogen over-accumulation independently from diet. In particular, two parameters found upstream of other modifications are pivotal to explaining glycogen supercompensation.

The first is a stable decrease in ATP and P-Cr levels, along with the net loss of purine nucleotides that occurs during the rest period in muscles stimulated for 24 hours. We believe that the net loss of purines is an indication that the muscle had entered long-term fatigue, preventing recovery of the cellular energy charge [36]. This also correlates with the sustained phosphorylation of AMPK during glycogen recovery and supercompensation. In addition, we report an increase of the content of AMPK1α subunit 24 hours after the stimulation period, contributing to enhanced AMPK activity, in agreement with previous studies on CLFS [8]. Activation of AMPK increases glucose uptake by translocating GLUT4 to the sarcolemma [37] and enhances fatty acid uptake and oxidation and mitochondrial biogenesis [9], which is consistent with the previously reported effects of long-term CLFS [38]. In this context, glucose could be spared from glycolysis and directed towards the synthesis of glycogen, favouring supercompensation.

The second important upstream factor is an enhanced capacity for glucose phosphorylation, due to increased HK activity, likely ascribed to HK-2. In addition to the increased *hk2* expression reported here, the contribution of HK-1 to total HK activity in muscle lies at around 6–10% [39] and previous studies have shown that muscle contraction increases HK-2 activity and gene expression, without changes in HK-1 [40], [41]. Fueger and Wasserman [4], [5] demonstrated that glucose phosphorylation becomes the limiting component in muscle glucose uptake during hyperinsulinaemia or exercise. Moreover, a delayed increase in HK in the group subjected to 1 hour of CLFS, which occurred when glycogen synthesis had finished and other modifications had already faded, did not lead to supercompensation, in agreement with previous studies [42]. Taken together, these findings highlight the crucial role of hexokinase and the need for coordination with other events leading to glycogen supercompensation.

In contrast to the above, glycogenin content remained unchanged during exercise and rest. Therefore, the increase in glycogen content in this model is due to an increase in the size of preexisting molecules, rather than an increase in their total number, correlating with a previous study of glycogen resynthesis [43], but arguing against a role in glycogen supercompensation, as reported elsewhere [16].

Furthermore, supercompensation could not be explained by an increase in the total activity of GS or a decrease in GPh. We only observed a decrease in total GS activity immediately following 1 hour of CLFS, with no accompanying change in protein content. This phenomenon has been reported after intense contractile activity [44] but is still not fully understood. On the other hand, although there was a close inverse correlation between the phosphorylation of sites 1a, 1b, 2+2a, 3a and 3b with glycogen content and mGS activity in all groups, as previously reported [45]–[47], after 1 hour of rest, in the 24-hour CLFS group we recorded decreased phosphorylation of sites 1b and 3a and increased GS fractional activity despite the presence of basal glycogen levels. To our knowledge, this is the first study to have reported such a disconnection, which can be attributed to regulation of site 3a phosphorylation, a site that is known to control GS activity [2]. The participation of site 1b in this model is not clear, since its role in GS regulation is not well understood. However, mGS is known to be translocated to different cell structures or compartments [17], [44], [47], [48]. In particular, Prats et al. [49] reported that during glycogen resynthesis, mGS is redistributed into intramyofibrillar and intermyofibrillar compartments, depending on the phosphorylation of site 1b and site 2+2a, respectively. Thus, dephosphorylation of mGS site 1b and hyperphosphorylation of site 2+2a suggests that mGS is involved in the elongation of the intermyofibrillar glycogen in the supercompensated pool. Overall, site 2 phosphorylation remained unchanged under all experimental conditions despite increased GS activity, in accordance with Lai et al. [46]. This might be attributed to the balance between regulatory signals working on this site, rather than a lack of control. One candidate is AMPK, since it was found to be hyperphosphorylated in the present study and has been suggested to be a mGS site 2 kinase *in vivo* [7].

In parallel, there was a sustained decrease in the mGPh activity ratio after 24 hours of CLFS. This suggests a role for mGPh in allowing glycogen turnover towards synthesis, and further maintaining supercompensation *in vivo*, as reported in other models such as *ex-vivo* muscles and cell culture [13], [50]. Protein phosphatase 1 may potentially control the differential regulation of mGS and mGPh phosphorylation [51]. Further research is required to fully understand this role.

Another important and novel outcome of this study is that newly synthesized glycogen becomes transiently less branched, returning to its basal structure when glycogen turnover is restored, regardless of changes in GBE. This phenomenon is similar to that found in various rare genetic conditions, such as Lafora disease [27]. Moreover, although glycogen is known to inhibit AMPK phosphorylation [52], [53], poorly branched polyscacharydes are less potent inhibitors *in vitro* [54], further correlating with our observations *in vivo*. We also demonstrated that poorly branched glycogen is less efficient substrate for GPh, similar to the enlarged glycogen model proposed by Melendez et al. [55]. Therefore, differences in branching could explain the discrepancies in exercise performance found in studies reporting improvements [56] or lack of any effect [57] related to increased muscle glycogen content. To maximize exercise performance, we propose adapting the timing of pre-exercise training strategies, such that glycogen is supercompensated during recovery, but branching is allow to revert to normal.

In summary, our results show that glycogen supercompensation, comprising enlargement of pre-existing glycogen molecules, is a phenomenon involving a coordinated chain of steps at two levels: 1) Upstream of the glycogen molecule, in association with depleted cell energy charge and increased capacity for glucose phosphorylation. 2) At the glycogen molecule level, comprising increased activity of mGS due to the dephosphorylation of site 3a, even at basal glycogen levels, and a stable decrease in the activity ratio of mGPh during the supercompensation period. However, early supercompensated glycogen comprises a store of enlarged poorly branched molecules, which are less efficient as a GPh substrate.
